# Development of NIST Atomic Databases and Online Tools

**DOI:** 10.3390/atoms8030056

**Published:** 2020

**Authors:** Yuri Ralchenko, Alexander Kramida

**Affiliations:** National Institute of Standards and Technology, Gaithersburg, MD 20899, USA

**Keywords:** atomic databases, standard reference databases, atomic spectroscopy, collisional-radiative modeling, laser-induced background spectroscopy (LIBS), bibliographic databases

## Abstract

Over the last 25 years, the atomic standard reference databases and online tools developed at the National Institute of Standards and Technology (NIST) have provided users around the world with the highest-quality data on various atomic parameters (e.g., level energies, transition wavelengths, and oscillator strengths) and online capabilities for fast and reliable collisional-radiative modeling of diverse plasmas. Here we present an overview of the recent developments regarding NIST numerical and bibliographic atomic databases and outline the prospects and vision of their evolution.

## 1. Introduction

The development of atomic databases at the National Institute of Standards and Technology (NIST) is an integral part of the NIST Standard Reference Data (SRD) Program. As follows from its clearly defined responsibilities under 15 U.S. Code §290 [1] to collect data, evaluate data, and publish high-quality SRD, NIST creates SRD products, of which atomic databases containing evaluated and recommended atomic data are an important component.

The creation and development of atomic databases is closely connected to the atomic physics program, and this synergy is extremely beneficial for both efforts. The atomic spectroscopy research at NIST goes back more than 100 years. To the best of our knowledge, the first results were published in 1913. Since then, NIST has become the leading research institution in the United States for the analysis of such atomic structure parameters as energy levels, oscillator strengths, and transition probabilities. This research is very active nowadays, and our experimental program is supported by an unparalleled combination of light sources (e.g., sliding sparks or an electron beam ion trap) and unique spectrometers ranging from infrared Fourier Transform Spectrometers (FTS) to a 10.5-m vacuum ultraviolet spectrometer and to an X-ray cryogenic transition-edge-sensor spectrometer that generate high-precision spectroscopic data from hard X-rays to the infrared parts of electromagnetic spectrum. Moreover, involvement of the NIST scientists in genuine world-class experiments allows them to gain experience in analysis and uncertainty evaluations of accurate spectroscopic data for atoms and ions.

The evaluation of atomic data at NIST started with the classical work of Charlotte Moore-Sitterly in the mid-1940s [2], which culminated in her famous compilation of atomic energy levels [3-5]. Thereafter, this activity has become one of the most visible and important components of research in the Atomic Spectroscopy Group. Although the actual number of physicists involved in data evaluation varied significantly over decades, it has never been neglected and the new data compilations are released and published on a regular basis.

After invention of the World Wide Web (WWW), the natural step for atomic databases was to utilize numerous advantages of WWW and develop the online versions of the NIST atomic data collections. This work was initiated in mid-1990s under the guidance of W.C. Martin and rapidly resulted in the creation of a number of atomic databases, from the most general ones, such as the Atomic Spectra Database (ASD) [6], to the more specialized (i.e., the Chandra database for only four elements of interest for the NASA Chandra X-ray telescope). With time, it was found that to provide users with more convenient access to the whole set of the available data, it is beneficial to join all sets of the evaluated atomic data into ASD. Therefore, over the last 10+ years, our efforts for numerical evaluated atomic data have been solely focused on ASD.

Since the mid-2000s, much effort was put into development of other atomic-physics-related databases and tools. To this end, in collaboration with the Lawrence Livermore National Laboratory we developed an online version of the highly popular and versatile collisional-radiative code FLYCHK [7], which allows extremely fast calculation of light emission from practically any plasma. Additionally, a new generation of NIST atomic bibliographic databases was established.

Below we provide an overview of the NIST atomic databases and online tools. We pay special attention to the data selection process and describe numerous features of our databases. Some examples of online calculations are provided as well.

## 2. Data Selection

The main principle of the NIST atomic databases is internal consistency. The second important requirement follows from the “standard” nature of these databases: all the data must have critically evaluated uncertainties. There are many other atomic databases providing useful data, but none of them satisfy these requirements. It should be noted that, although a significant portion of the ASD data taken from old legacy sources does not have explicitly shown uncertainties, they were evaluated in the original compilations and can be retrieved from the references quoted in ASD. For such data, a rough estimate of uncertainty is implied by the number of significant figures displayed in the database.

Providing critically evaluated internally consistent data imposes severe limitations on responsiveness of NIST databases to new publications. For the last several decades, about 500 new papers containing atomic data have been published every year. The NIST Atomic Spectroscopy Group has a set of specially designed bibliographic databases [8-10], in which these new publications are stored with a small delay of a few months or even weeks after the paper is published. However, getting the published data into our main numerical information system, ASD [6], is much more time-consuming.

Most of the published articles contain fragmentary studies covering small subsets of data on certain atomic spectra. For example, one or a few atomic transitions could be precisely measured using laser spectroscopy in a cold atomic trap or by other high-precision techniques. Within its scope, such a study provides valuable data on energy levels and transition frequencies. However, different transitions studied by other authors may (and usually do) provide alternative data on the same parameters, which often disagree with the new measurements. To provide a self-consistent set of data for each spectrum, it is necessary to analyze the new measurements in the broad context of all other available data. Resolving contradictions between different sources of data is very difficult and sometimes requires extensive analytical work. Sometimes it requires making new measurements and/or theoretical calculations. A typical example is the recent critical compilation of Cu II spectral data [11], which took several years to complete. As a result, most of the data sets in ASD are missing the latest new determinations. We are constantly working on updating and extending the database, but on average, only a few spectra are added or updated in each new version of ASD released every year.

The methods used in our critical compilations have been explained in a number of publications, notably by Wiese [12], Reader [13], and Kramida [14]. To ensure traceability, we normally do not average data available from multiple sources. Instead, we select the “best” data, which means those that are consistent with other data and have the smallest uncertainties. This selection is usually non-trivial, since many data are published without strictly defined uncertainties for each determined value. This is especially true for theoretical data, which constitute about half of all data on transition probabilities in ASD. Until now, only about 2% of all theoretical papers provided estimated uncertainties for the calculated quantities. Only in recent years did we start to see an increasing number of theoretical studies using the NIST methodology to estimate uncertainties of their results. If this positive trend continues, it may make future data compilations faster and easier to do.

It should be noted that atomic theory continues to lag far behind the precision of experimental measurements in most spectra. Hydrogen- and helium-like ions are rare exceptions, where modern theoretical calculations can compete with experimental precision. Calculations of radiative transition rates and collisional cross-sections are especially difficult. In most theoretical studies, only a tiny fraction of the strongest transition rates involving low or moderately excited energy levels are calculated with a precision of a few percent or smaller. Quasi-random errors increase very fast with decreasing transition strength [14]. For atoms and ions with medium or large number of electrons (20 or greater), a common case is that a vast majority of the calculated results have large uncertainties (greater than a factor of two and up to several orders of magnitude). Rapid development of theoretical methods notably improved the accuracy of calculations in several types of atomic systems (e.g., atoms/ions with one electron outside a closed shell or one hole in a closed shell), but even for these systems the modern theory cannot handle highly excited and autoionizing states with adequate precision. Thus, analysis of observed spectra and accurate prediction of various atomic properties remain daunting tasks integral to our data evaluation process.

As of mid-2020, the data in ASD have been taken from about 3000 papers or other sources. Of these references, about 2000 are for energy levels and spectral lines (wavelengths, intensities, classifications), while the rest are for transition probabilities. At the same time, the NIST atomic bibliographic databases [8-10] contain about 34,000 references to published articles. About 26,000 of these articles contain numerical data on energy levels, spectral lines, and transition probabilities of atoms and atomic ions (the rest are either papers on line broadening, review articles, or descriptions of relevant experimental or theoretical methods or codes). From these numbers, one can see that only about 10% of all published data are incorporated in ASD. Thus, the bibliographic databases are a very important resource for researchers looking for data.

The users seeking the latest and greatest data should not completely rely on the data sets displayed in ASD. They should use the links to “current literature” on each spectrum displayed at the top of ASD output and browse through the papers published after the date of the primary data source indicated in the ASD output. These links are carefully designed so that they retrieve the most complete lists of references either on energy levels, spectral lines, or transition probabilities.

With each new release, some atomic data sets in ASD are being replaced or corrected. When this happens, the previous version of the data becomes inaccessible to online users. However, starting with ASD version 5, we have kept copies of each released version of ASD stored on our internal server and can provide the old data if requested by users.

## 3. Atomic Spectra Database

The NIST Atomic Spectra Database is the world’s largest source of evaluated and recommended data for atomic parameters such as energy levels, spectral lines, radiative transition probabilities (and oscillator strengths), and ionization potentials. As of mid-2020, ASD contains 112,142 energy levels, 280,135 spectral lines, 118,203 transition probabilities, and 6019 ionization potentials. In the past few years, ASD received about 60,000 distinct data requests per day on average. The spectroscopic data are available for elements from H to Ds (Z = 110), although not for all ions of heavy species. It is certainly not unanticipated that the neutral and low-charge ions are best represented in ASD since very high temperatures and/or energies are required to produce highly-charged ions.

**Figure 1.**
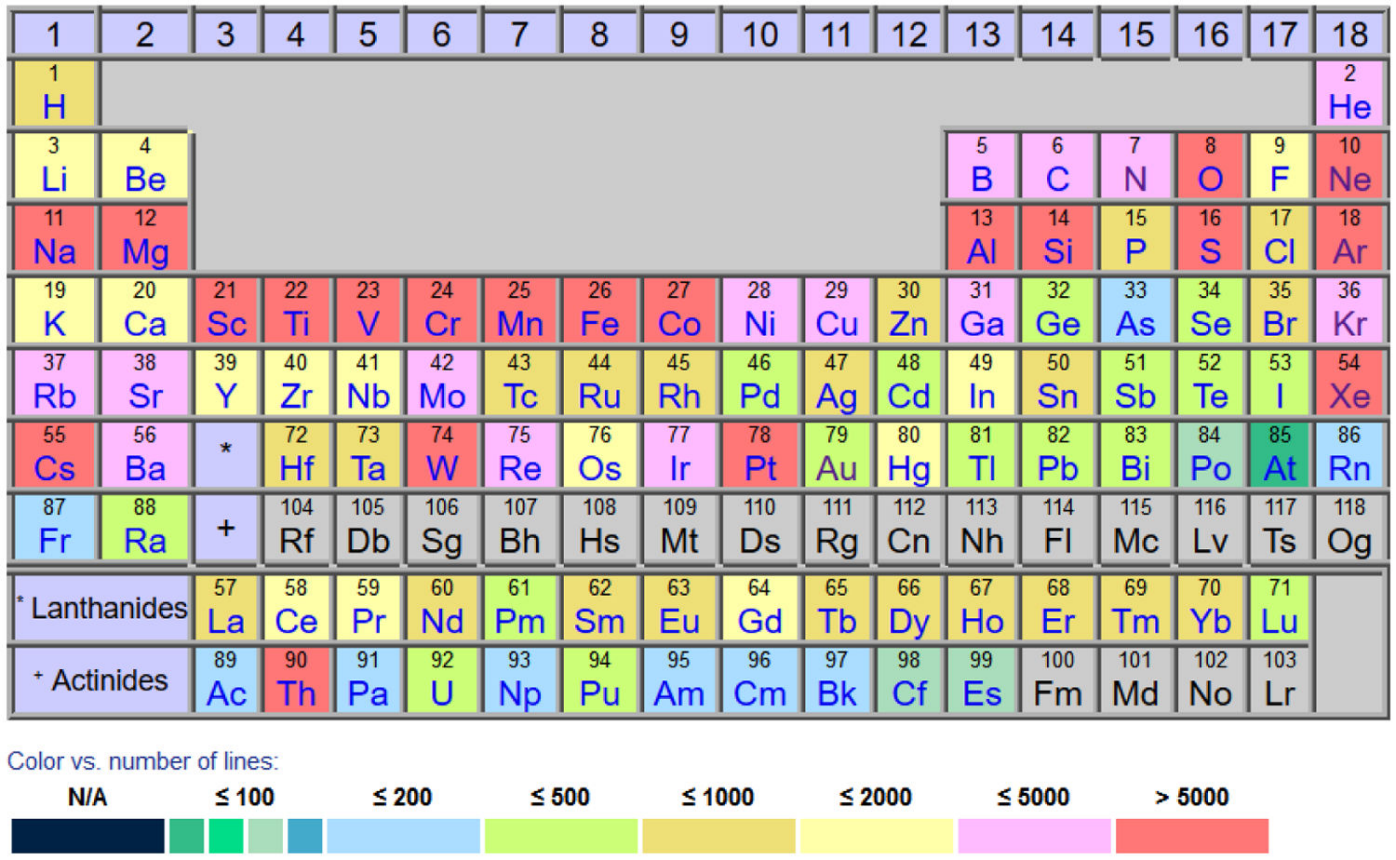
Contents of ASD version 5.7.1 (released in October 2019) for spectral lines.

Figure 1 presents the current contents of ASD for spectral lines. Different colors correspond to different numbers of lines for a particular element. As one might expect, it is the low-Z elements that are well covered within ASD. Indeed, such important astrophysical and terrestrial spectroscopy elements as O, Si, and Fe have 5913, 5696, and 29,609 spectral lines, respectively, in the database. As for the heavy elements, W (14,703 lines) and Pt (9136) and Th (20,143) are represented very well too: the former due to its importance for the magnetic fusion program, and the latter due to their crucial role in the calibration of spectroscopic instruments on astronomical telescopes. As for the energy levels, the iron-group elements have the largest number of data available varying from 2229 for V to 4429 for Fe.

ASD offers a number of options for search and selection of data. In addition to rich graphical tools to explore the output (see below), a user can perform a search for specific ions or isoelectronic sequence in different spectral ranges, look for observed or Ritz wavelengths, ask for data on transition probabilities including forbidden lines, set limits on level energies or transition strengths, modify the output units, and so on. Importantly, in most cases the output contains complete information on level quantum numbers, including configurations, terms, and total angular momentum. It is essential that data uncertainties for wavelengths are displayed by default. The output can be produced in either HTML or various ASCII formats (fixed column width, tab-delimited, or CSV) for easy downloads, and wavelength-ordered or *LS*-multiplet-ordered tables are generated on demand. Many of the output options for spectral lines are also available for the level output.

Since the first online version of ASD, significant efforts were put into the development of advanced graphics and visualization options that would enhance users’ experience and facilitate analysis of the ASD data. Here we briefly discuss the main graphical options and tools available for ASD users.

### 3.1. Grotrian Diagrams

The Grotrian diagrams were introduced in the early 1930s and have been available in ASD since 2005. They depict the energy structure, ionization energies, and radiative transitions of an ion using proper energy scales. The interactive diagrams of ASD provide immediate access to a wealth of information on levels and lines. Clicking on a particular level not only highlights the levels and all possible radiative transitions from and into it, but also displays almost all important physical data including its energy, configuration, atomic term, *J*-value, and so on. With the “isolate” button, a user can remove all other levels and lines not related to the selected level and thus better explore possible connections of the level. Then, by clicking on a line, a user can easily see the upper and lower levels of this radiative transition with all related data, and the corresponding wavelengths (both observed and Ritz), transition probability, oscillator strengths, and so on. Again, the isolation option is available for lines, too. There is a multitude of other options, including selection of particular configurations, subconfigurations, *J*-values, and multiplicities, setting a range of transition probabilities for the output, zooming in and out, and others.

**Figure 2.**
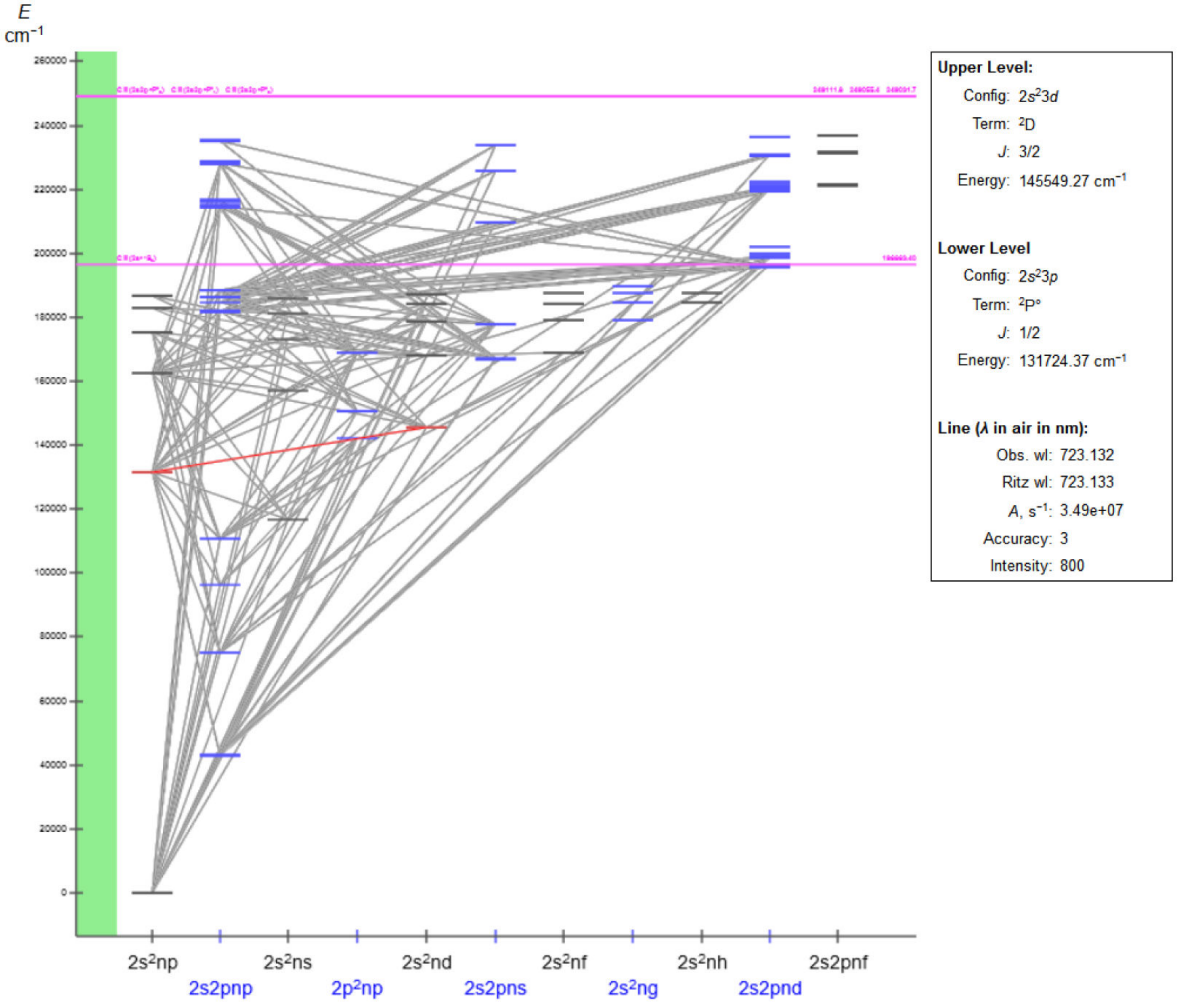
Grotrian diagram for C II. The box represents the available data for the selected spectral line (in red).

An example of a Grotrian diagram for the C^+^ ion is given in Figure 2. The atomic levels are grouped into series according to the *nl* values of the outermost electron. The ionization limits for different core configurations are shown by horizontal magenta lines. Additionally, the autoionizing levels can be easily identified. Finally, the box on the right shows detailed information on the selected spectral line, which is highlighted in red.

### 3.2. Line Identification Plot

This simple option was added on a direct request from ASD users who were interested not in relative line intensities but rather in positions of spectral lines. Therefore, selecting this option would produce a bar-like graph, where all spectral lines of each ion have the same height, which is different from other ions. This allows one to easily recognize the contributions from different ions to the measured spectra, although a more detailed analysis should utilize more sophisticated approaches (see below).

### 3.3. Saha-LTE Spectrum

Saha-LTE (i.e., local thermodynamic equilibrium) spectra can be generated directly from the ASD input of the ion range for a specific element, spectral range, electron temperature and density, and ion temperature for Doppler broadening. A more advanced tool for the same kind of online calculations was developed more recently (see Section 3.4), but it was decided to keep the Saha-LTE service for legacy purposes. Both tools use the same physical model (i.e., Saha-LTE) but differ in flexibility of interactive options, as explained in the next subsection.

### 3.4. LIBS Database

In some cases, the fundamental atomic data stored in ASD can also be used to derive spectral characteristics of relatively simple equilibrium plasmas. As is well known, in the most general case of arbitrary plasma parameters, one has to implement some kind of a collisional-radiative model in order to determine the atomic state populations and the ensuing spectra [15,16]. This requires utilization of large sets of collisional parameters (e.g., cross-sections or rate coefficients) that are not available in ASD. However, the ionization distributions, level populations, and spectral emissivities in optically thin low-temperature high-density equilibrium plasmas can be determined from analytical Saha–Boltzmann (or LTE) formulas that only depend on ionization energies, level energies, their statistical weights, and radiative transition probabilities. Fortunately, these are precisely the physical parameters that ASD contains, and thus Saha–Boltzman spectra can be generated online easily and quickly.

Such Saha-LTE distributions are typical for low-temperature, high-density plasmas [17]. One of the most known examples of such plasmas is produced in laser-induced breakdown experiments. Here, a short laser pulse evaporates a tiny amount of a sample material, heating it to about 1 eV. A relatively new analytical technique, the laser-induced breakdown spectroscopy (LIBS) is used primarily to determine the compositions of elements in various materials, for instance, rock and minerals on Mars. Currently, there are literally hundreds of LIBS-related papers published annually, thereby exemplifying importance of LIBS research in contemporary science and technology.

The LIBS database at NIST [18] allows a user to calculate the Saha-LTE spectrum for an arbitrary combination of chemical elements that can be found in ASD. Of course, such a calculation requires, as mentioned above, energy levels and transition probabilities, which, unfortunately, are not available for all spectral lines in our database. Nonetheless, since LIBS is typically used for low temperature plasmas with low-charge ions, this situation is rectified by the fact that such ions are quite well presented in ASD.

The actual list of physical parameters to be used on the LIBS input page includes the following:

- List of chemical elements with their percentages in the mix (the total sum must be 100%);
- Range of wavelengths;
- Choice of vacuum or vacuum+air output for the wavelengths;
- Wavelength units;
- Spectral resolution;
- Electron temperature;
- Electron density.

The advanced options also include the maximum ion charge and the minimum relative line intensity (with regard to the strongest line) to be included in calculations.

The output page presents a detailed graph of the total spectrum along with the original data table in a number of formats. This output is fully dynamic and thus allows on-the-fly modifications and recalculations of the spectrum via varying the input parameters. A user can not only download the calculated spectrum but also upload a file of the measured spectra to have a very convenient comparison of the LIBS-calculated and original data.

As mentioned above, the old Saha-LTE graphical tool accessible via the line section of ASD remains functional and provides essentially the same kind of modeling. The LIBS database interface was tailored for typical needs of LIBS researchers. It is more flexible than the Saha-LTE tool in its ability to model arbitrary mixtures of elements, to provide online comparisons with experimental spectra, and in the variety of interactive options in plots. However, it has no provisions for the modeling of spectra of specific isotopes, which is available in the Saha-LTE tool. Another difference is specific to the hydrogen spectrum, where the LIBS database uses only the unresolved lines corresponding to transitions between centers of gravity of fine-structure levels with certain principal quantum numbers, while the Saha-LTE tool allows for a choice between displaying spectra for unresolved or resolved fine structure.

## 4. Online Plasma Emission Modeling

**Figure 3.**
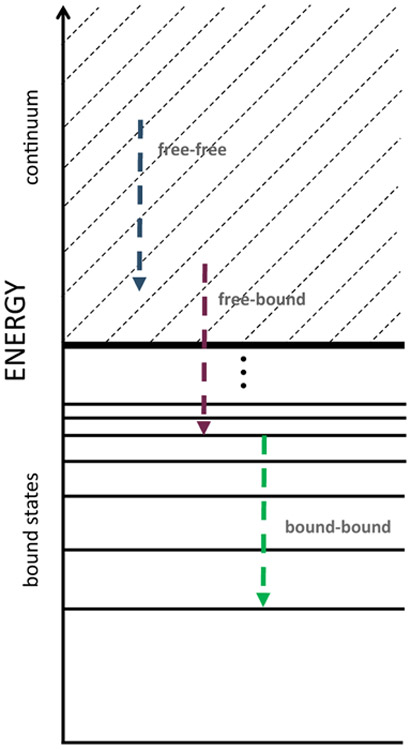
Energy scheme for free-free, free-bound, and bound-bound electron transitions.

Spectroscopic diagnostics of plasmas utilize a variety of techniques and approximations. It is customary to separate plasma emission into free-free (bremsstrahlung), free-bound (radiative recombination), and bound-bound (line emission) parts corresponding to different paths of electron movement in the energy space (Figure 3). The bremsstrahlung intensity is largely determined by the mean charge of the ions in the plasma and the electron temperature. As for the bound-free and bound-bound transitions, in addition to the purely atomic parameters (i.e., spontaneous transition probability for the latter and radiative recombination cross-section for the former), the intensity depends on the population of the initial state, which in turn is affected by all possible important physical processes that can bring an electron to that particular atomic state. In order to determine the corresponding populations, it is customary to make use of advanced collisional-radiative models that use (time-dependent) rate equations for this task.

FLYCHK [7] is a time-dependent collisional-radiative code for calculation of level populations, plasma emissivities, intensities, radiative power losses, and opacities for elements from H to Au (Z = 79). Its original goal was to provide experimentalists with an extremely fast (a typical calculation takes only a few seconds) and reliable tool that can assist in diagnostics of various plasmas. While the methods and techniques used in FLYCHK simulations are best applicable to complex ions in high-temperature mid- to high-density plasmas, it has been successfully used for relatively cold and diluted plasmas as well. Currently the FLYCHK code at NIST has more than 1200 registered users from dozens of countries and laboratories, and it is used many times every day.

FLYCHK can calculate the ionization balance and emission/absorption spectra in steady state and transient plasmas, including a variety of plasma effects. For instance, both electron temperature and electron, ion, or mass densities can have arbitrary dependencies on time. Moreover, the code allows for a mixture of elements, opacity effects, an arbitrary electron energy distribution function and radiation field, an ion temperature different from that for electrons (to better describe Doppler broadening), ionization potential lowering in different approximations (e.g., Stewart–Pyatt and Ecker–Kroll), and other effects. A typical set of the output parameters includes plasma mean ion charge and ionization distributions; radiative losses (bound-bound, free-bound, free-free, and total); and intensities, emissivities, opacities, and optical depths.

**Figure 4.**
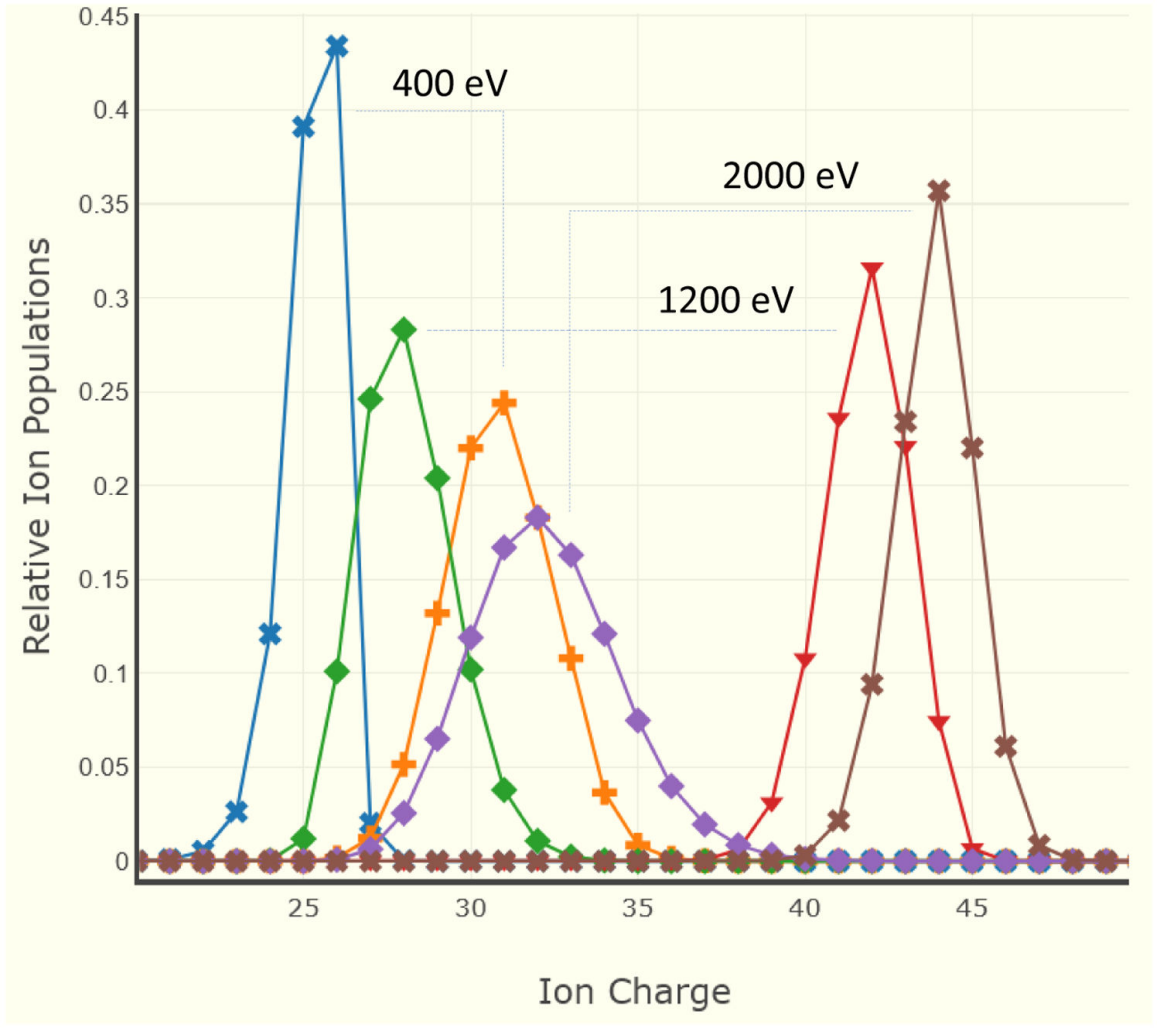
FLYCHK ionization distributions for a steady-state Xe plasma at electron temperatures 400, 1200, and 2000 eV, and electron densities 10^18^ and 10^24^ cm^−3^. The ionization curves for higher densities are shifted to the right due to enhanced ionization via excited states.

An example of the graphical output for FLYCHK calculations is given in Figure 4. Here, the task was to determine level populations and other parameters for a steady-state Xe plasma at electron temperatures of 400, 1200, and 2000 eV, and electron densities of 10^18^ and 10^24^ cm^−3^. This calculation took only 7 s on a very modest PC. In this figure, the ionization curves for higher densities are shifted to the right due to the well-known effect of enhanced ionization via excited states.

**Figure 5.**
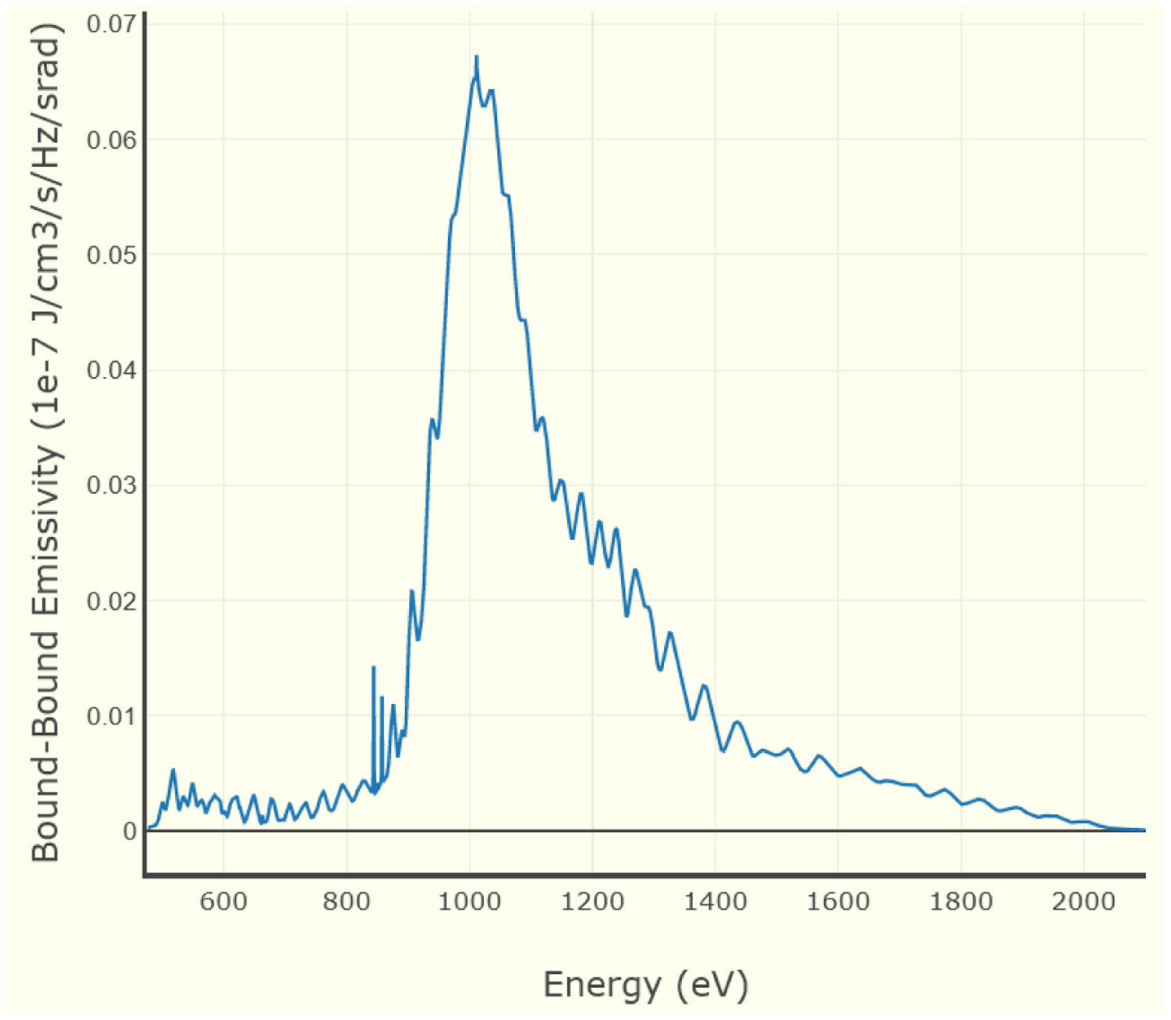
Bound-bound emissivity for a steady-state Xe plasma at 2000 eV and 10^18^ cm^−3^.

One of the most important features of FLYCHK is its capability to generate spectra. Figure 5 presents the bound-bound emissivity calculated for a steady-state Xe plasma at 2000 eV and 10^18^ cm^−3^ in the spectral range of 500 to 2000 eV. Such data allow direct comparison with the measured spectra and thus provide an important tool for diagnostics of plasmas.

## 5. Collaborations

The development of modern online atomic and molecular databases is more and more becoming an international collaborative effort rather than a local project. There are many atomic and molecular databases varying in data coverage, data quality, and underlying database management systems. Nonetheless, the programmatic, and in particular, data exchange issues are rather common across the field and thus, interactions between database developers are highly beneficial. The NIST ASD team tries to develop and utilize such interactions to their fullest extent, and the benefits of collaborations (e.g., FLYCHK development) are numerous and visible.

One of the examples of such fruitful collaboration is provided by the Virtual Atomic and Molecular Data Centre (VAMDC) [19]. The current status of VAMDC is described in detail in a separate paper [20] of this Special Issue. NIST was a part of the VAMDC project from its very beginning, and ASD is one of the many databases that can be directly queried from the VAMDC portal [21]. Additionally, NIST scientists were actively involved in development of the XML Schema for Atoms, Molecules and Solids (XSAMS), which has become the standard for data exchange within VAMDC. This collaboration has significantly affected our approach to database development and it continues to positively influence our present and future activities.

## 6. Conclusions

The development of atomic databases and online tools represents a very important activity at NIST. This work relies on rich experience gained over decades of production of evaluated and recommended atomic data. Modern research in atomic physics is clearly shifting away from “classical” tasks on identification of atomic spectra; nonetheless, it is hard to overestimate the value of precise atomic data for different types of spectroscopic research, from cold atoms and atomic clocks to industrial plasmas to astrophysics and extremely hot fusion plasmas. The Atomic Spectroscopy Group at NIST is fully committed to continuation of this important work of high value for the atomic physics community.

The content of ASD is gradually expanding to ensure more complete coverage of atomic spectra. We will continue to update ASD in the form of new yearly releases. The current work includes a compilation of satellite spectra of all Li-like ions, which are important for plasma diagnostics in astrophysical and laboratory high-temperature plasmas. Spectra of atoms and ions of the iron group elements are also in our current plans. Priorities for our work on ASD are greatly influenced by user requests that we receive via contact links provided in the output pages of ASD. In parallel with data expansion and correction, we will continue improving the supporting software, which also relies to a large extent on interactions with users. The bibliographic databases on atomic spectra [8-10] will continue to be updated at least every week, ensuring complete coverage of current and past literature. Other online resources described in the article are also continuously being developed and improved. In particular, the database of collisional and radiative data used in the FLYCHK modeling is being upgraded with new data, and we will continue to improve its spectral modeling features.

## Abbreviations

The following abbreviations are used in this manuscript:

ASD: Atomic Spectra Database
EBIT: Electron Beam Ion Trap
FTS: Fourier Transform Spectroscopy
LIBS: Laser-Induced Breakdown Spectroscopy
LTE: Local Thermodynamic Equilibrium
NIST: National Institute of Standards and Technology
SRD: Standard Reference Data
